# Acute localized exanthematous pustulosis caused by cefoperazone and sodium sulbactam*

**DOI:** 10.1590/abd1806-4841.20165182

**Published:** 2016

**Authors:** Yan-Jing Qu, Shu-Bin Jin, Xiang-Chun Han, Li-Qiang Zheng

**Affiliations:** 1 The 251st Hospital of Chinese PLA – Zhangjiakou city, China; 2 HanDan Central Hospital - Handan city, China; 3 The First Affiliated Hospital to Hebei North University - Zhangjiakou city, China; 4 Chinese People's Liberation Army General Hospital - Beijing, China

**Keywords:** Acute generalized exanthematous pustulosis, Cefoperazone, Sulbactam

## Abstract

Acute localized exanthematous pustulosis is a localized variant of acute generalized exanthematous pustulosis, which is characterized by the eruption of multiple scattered pustules following drug administration. A 72-year-old woman presented with multiple erythematous pustules on her face, which had appeared two days after using cefoperazone and sodium sulbactam. Histopathological findings showed subcorneal pustules and mixed inflammatory cell infiltration in the dermis. The pustules resolved within about two weeks after the patient discontinued the antibiotics. This report discusses the case of a woman with a cutaneous drug reaction consistent with acute localized exanthematous pustulosis that occurred after cefoperazone and sodium sulbactam were administered.

## INTRODUCTION

Acute generalized exanthematic pustulosis (AGEP) is a rare but well-known cutaneous reaction pattern, mostly caused by drugs.^1^ This condition is characterized by a generalized rash and sterile, disseminated, sometimes coalescing, subcorneal pustules on an erythematous background. The reaction is self-limited once the causative drug is withdrawn. A localized variant of AGEP, acute localized exanthematous pustulosis (ALEP), occurs rarely.^2^ Previous studies have found that drugs like amoxicillin, levofloxacin, paracetamol, ibuprofen, finasteride and piperacillin/tazobactam may induce ALEP attacks.^3-8^ Herein, the authors describe a case of ALEP associated with cefoperazone and sodium sulbactam.

## CASE REPORT

**Figure 1:**
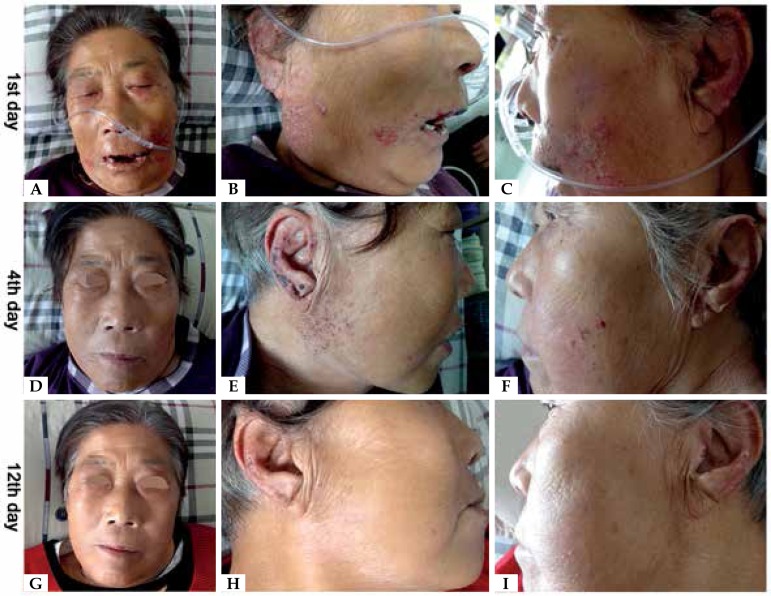
(a)-(c) Multiple pustules underlying erythema, distributed symmetrically, involving her cheeks, auricles and lips with erosion and crust on day 1.(d)-(f) Pustules subsided rapidly on day 4 after the suspicious drug was withdrawn.(g)-(i) The rash disappeared completely on day 12

A 72-year-old woman was admitted to our hospital due to pneumonia, involving a fever of 38.0ºC, a cough and yellow sputum, and nausea without vomiting. She was treated with cefoperazone and sodium sulbactam for two days (3g, b.i.d, iv gtt). Later, she had a higher fever of 40ºC and complained of an acute outbreak of multiple pustular lesions, affecting her face. Physical examination revealed multiple pustules underlying erythema, symmetrically involving her cheeks, auricles and oral lips with erosion and crust (Figure 1). Given the possible allergic reaction to the drug and the extent of renal dysfunction (Serum creatinine 251.3umol/L and BUN 13.1mmol/L), over the following seven days, cefoperazone and sodium sulbactam were discontinued. To prevent secondary infection, topical treatment with phudicin cream was employed. The acute eruption subsided gradually and resolved completely within 10 days, followed by punctiform pigmentation (Figure 1). She had a medical history of hypertension (20 years' duration), treated with enalapril maleate (10mg, q.d, p.o); and of cerebral infarction (5 years) without timely treatment. Her blood pressure was well-controlled while barylalia sequelae persisted. No history of herpes zoster or psoriasis was recorded.

**Figure 2:**
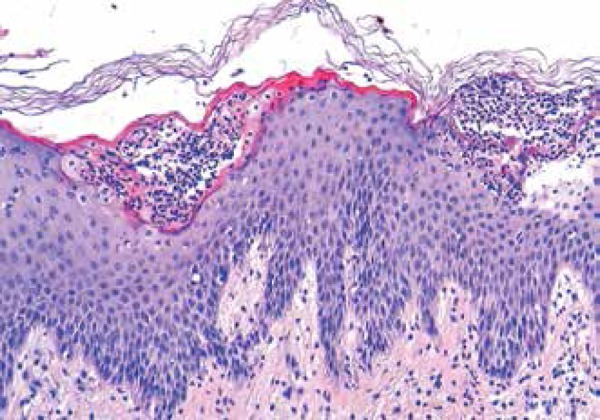
Histopathological features revealed subcorneal pustules were accompanied by a slight psoriasiform acanthosis in association with spongiosis. (HE:×200)

A lesional biopsy specimen from the right auricle revealed a slight psoriasiform acanthosis in association with spongiosis, and infiltration of the epidermis by neutrophils, resulting in the formation of subcorneal pustules, consistent with AGEP (Figure 2).

**Figure 3:**
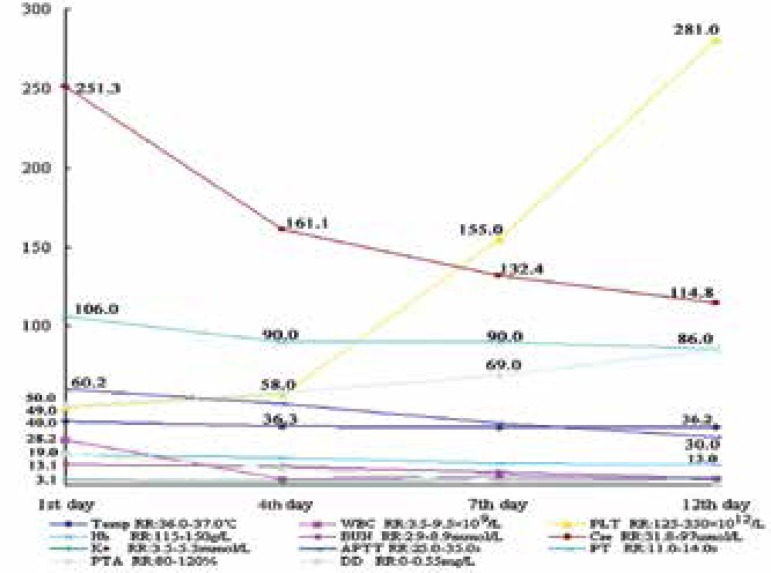
Laboratory data from the 1st, 4th, 7th to 12th days were recorded

Laboratory data from the 1st, 4th, 7th to 12th days were significant (Figure 3). The results of viral cultures and PCR, as well as bacterial and fungal cultures of skin lesions, proved negative.

## DISCUSSION

AGEP is a severe, usually drug-related, skin eruption characterized by acute formation of sterile pustules on an erythematous background, entailing an associated fever and neutrophilia.^1^ Commonly associated drugs include macrolide antibiotics, aminopenicillins, quinolones, hydroxychloroquine, sulfonamides, terbinafine and diltiazem. On average, the onset of lesions varies from as early as 24 hours after therapy initiation, to 1 to 2 weeks following treatment. ^1,9^

Skin symptoms resolve rapidly within a few days without treatment. The rash is usually accentuated in the large folds. Mucous membrane involvement is rare, commonly mild, and generally restricted to one site, mostly the oral lips.

Histology shows: subcorneal pustules, intradermal pustules, or both; sometimes pronounced edema in the papillary dermis; and perivascular infiltrates consisting of neutrophils and sometimes eosinophils.^1^

The physiopathological mechanisms of AGEP remain uncertain, but drug-specific positive patch test responses and in vitro lymphocyte proliferative responses in patients with a history of AGEP strongly suggest that this reaction occurs by a drug-specific, T-cell-mediated process.^7,8,10^ IL-8 is likely responsible for recruiting neutrophils in the intraepithelial pustules. Recently, a relative increase in TH17 cell numbers and elevated levels of cytokine IL-22 in the peripheral blood of patients with AGEP was shown. TH17 cells may increase IL-8 production by keratinocytes through the effects of IL-17 and IL-22.^7,10^

Some authors suggest the term ALEP for this possible variant of AGEP, because both the clinical and histopathologic findings of ALEP are similar to those of AGEP. The definition of ALEP was introduced by Prange *et al.*^2^ Over 15 ALEP cases have subsequently been reported.^2-8^

The differential diagnosis included varicella zoster virus infection, allergic contact dermatitis, infectious folliculitis and IgA pemphigus. However, a Tzanck smear, a PCR of the virus and bacterial cultures were negative. Notably, the lesions disappeared immediately after withdrawal of cefoperazone and sodium sulbactam. The team assumed that this was attributable to the antibiotic.

In this case, the patient exhibited a symmetrical, localized, pustular eruption on the face. Simultaneously, she experienced a transient fever, elevated white blood cells count with neutrophilia, thrombocytopenia, as well as hemostasis and hypokalemia disorders, and renal dysfunction. As soon as the drug was stopped, the rash showed a rapid resolution and accordingly, laboratory exams were within normal values. Recently, a similar case with lesion onn the upper thigh was reported following piperacillin/tazobactam use.^8^

As is widely known, the systemic adverse effects of cefoperazone and sodium sulbactam include gastrointestinal reactions (mild diarrhea, nausea and vomiting, etc.). The hematological system side effects consist of decreased neutrophils, hemoglobin, and platelets, hypoprothrombinemia and eosinophilia. Laboratory tests indicated transient increased AST(SGOT)/ALT(SGPT), alkaline phosphatase, BUN and creatinine and other side-effects, along with headaches, fever and chills. So far, ALEP associated with cefoperazone and sodium sulbactam has not been reported.

Indeed, given the temporal relationship between the administration of the antibiotic and the development of the skin disease and the histologic findings, the authors consider this to be an unusual type of AGEP, defined as ALEP induced by cefoperazone and sodium sulbactam.
